# Living the Sweet Life: How Does a Plant Pathogenic Fungus Acquire Sugar from Plants?

**DOI:** 10.1371/journal.pbio.1000308

**Published:** 2010-02-09

**Authors:** Nicholas J. Talbot

**Affiliations:** School of Biosciences, University of Exeter, Exeter, United Kingdom

Plant diseases are an important constraint on worldwide crop production, accounting for losses of 10–30% of the global harvest each year [1]. As a consequence, crop diseases represent a significant threat to ensuring global food security. To feed the growing human population it will be necessary to double food production by 2050, which will require the sustainable intensification of world agriculture in an era of unpredictable climate change [2],[3]. Controlling the most important plant diseases represents one of the best means of delivering as much of the current productivity of crops as possible. To accomplish this task, a fundamental understanding of the biology of plant infection by disease-causing agents, such as viruses, bacteria, and fungi will be necessary [1],[2].

Fungal pathogens can broadly be divided into two groups—the biotrophs and necrotrophs [4],[5]. Biotrophic pathogens are parasites that have evolved the means to grow within living plant cells without stimulating plant defence mechanisms [6]. This means that they are able to spread rapidly throughout plant tissue while, at the same time, diverting nutrients from the living plant to fuel their own growth at the expense of plant productivity. In contrast, the necrotrophic pathogens use toxins and depolymerising enzymes to kill and degrade plant cells, consuming the resulting products [7]. These modes of nutrition are highly distinctive and plants have evolved independent defence mechanisms to contend with such different pathogens [5],[7]. To compound this challenge to plants, some pathogens exhibit both types of nutrition, switching from biotrophic growth to a rapid killing of plant cells as disease symptoms occur [7]–[9]. Because of their rather sophisticated nature, biotrophic pathogens cause some of the most pervasive plant diseases, which are difficult to control. Powdery mildew of barley caused by *Blumeria graminis*, for instance, continues to be one of the most important temperate cereal diseases [10], while yellow and brown rust diseases cause significant losses to worldwide wheat production [11]. The spread of the UG99 strain of wheat stem rust, which is highly virulent against most elite cultivars of wheat grown around the world, throughout Africa and the Middle East, shows how vulnerable existing cereal production is to attack by these sophisticated plant parasites [12].

In order to grow, a plant pathogenic fungus must secure an organic carbon source from the plant. In most plant diseases, however, we have little idea of what constitutes the major carbon source for an invading fungus during growth in plant tissue. Fungi are osmotrophic organisms, which means that they proliferate in a substrate by secreting a large diversity of extracellular enzymes that depolymerise polymers, such as cellulose, lignin, proteins, and lipids and then deliver the resulting simple sugars, amino acids, and fatty acids into fungal hyphae by means of plasma membrane-localised transporters [8],[13]. It is clear from analysing the genome sequences of both plant pathogenic and free-living fungi that they possess large numbers of extracellular enzymes and transporter-encoding genes, although, as might be expected, extracellular enzymes appear more restricted in number in biotrophic species [14]. The transporters, which are of the major facilitator transporter family, allow fungi to grow on an extremely diverse set of materials and are one of the reasons why fungi can occupy such a large number of ecological niches.

How do biotrophic plant pathogens acquire nutrients efficiently from a living plant cell? To understand this process it is essential to understand how plant pathogenic fungi enter living plant tissue. A large number of plant pathogenic fungal species develop specialised cells called appressoria that are able to breach the outer cuticle of plants and thereby gain entry to epidermal cells [8],[9]. The plant cells are not ruptured in this process, but instead the fungus is able to invaginate the plant plasma membrane and grow within the apoplast—the space between the plant plasma membrane and the plant cell wall [13]. This allows the fungus to occupy intact, living plant cells and set up a specialised interface to allow sequestration of nutrients directly from host cells [13]. A study published in this issue of *PLoS Biology* [15] provides a significant advance in understanding the mechanism by which a plant pathogenic fungus is able to acquire nutrients in planta. *Ustilago maydis* is a biotrophic pathogen which causes corn smut—a disease that is characterised by production of tumours on the stems and leaves of maize plants and, ultimately, by the liberation of large numbers of black teliospores that allow the fungus to be disseminated to new maize plants [16]. Corn smut can be a serious disease in maize-growing regions of Mexico and the United States [16]. In the study, the authors identified a novel plasma membrane-localised sucrose transporter encoded by the *SRT1* gene, and have shown its contribution to fungal virulence. *SRT1* encodes a plasma membrane protein with 12 membrane-spanning domains and is unusual because, in contrast to the relatively broad spectrum hexose transporters previously identified and characterised in fungi, Srt1 appears to be specific for the transport of sucrose. The conclusions of this study are that sucrose, which constitutes the most abundant storage sugar within plants and the product of photosynthesis, is directly utilised by invading pathogens without the need for its extra-cellular degradation by fungal secreted invertases.

**Figure 1:**
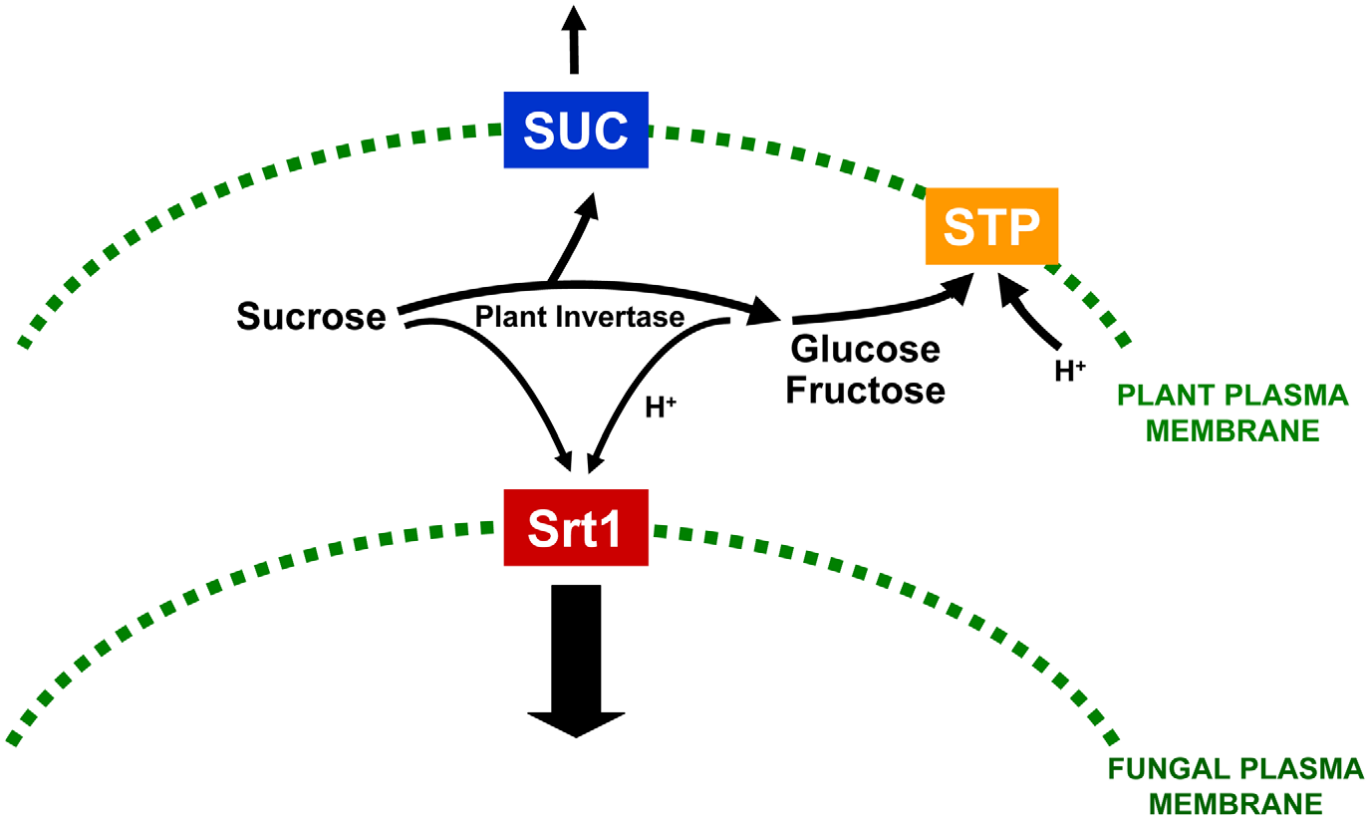
Efficient acquisition of sucrose by a plant pathogenic fungus. Model for the uptake of sucrose by the biotrophic plant pathogenic fungus *Ustilago maydis* based on a new study by Wahl and colleagues [15]. *U. maydis* possesses a high-affinity sucrose H+-symporter, Srt1, which is present in the plasma membrane of its invasive hyphae. The plant–fungus interface is established by invagination of the plant plasma membrane during intracellular invasive growth by the fungus. Srt1 competes for apoplastic sucrose with the plant SUC (or SUT) sucrose transporters [17],[18] and with plant invertases, which result in glucose and fructose generation. This reduces direct uptake of sucrose by plant cells or hexose uptake by the STP transporters, allowing the fungus to derive its primary carbon source from living plant cells without eliciting plant defence mechanisms. Adapted from Figure 8 of Wahl et al. [15].

The authors present a number of independent lines of evidence to support these conclusions. Srt1 was expressed in the yeast *Saccharomyces cerevisiae*, where they were able to study both its substrate specificity and affinity for sucrose. Srt1 is a sucrose-transporter with an extremely low K_M_ of 26±4.3 µM, which suggests that the fungus is able to out-compete plant sucrose transporters, such as the SUC family of energy-dependent H+ symporters that have an affinity for sucrose that is 20–200-fold lower than Srt1 [17],[18], and thereby gain an advantage in its proliferation within plant cells (Figure 1). The unusually high affinity of Srt1 for sucrose was measured by uptake experiments with ^14^C-labelled sucrose that also revealed its specificity for sucrose compared to other disaccharides, such as maltose, raffinose, and trehalose, as well as monosaccharides. Srt1 was also shown to be a plasma membrane–localised protein when expressed in *S. cerevisiae*, consistent with its transporter function [15]. Importantly, the authors also demonstrated that *SRT1* is expressed specifically during invasion of plant tissue and fails to be expressed by *U. maydis* when cultured away from a plant, either in the presence of sucrose or in the absence of glucose as sole carbon source. This suggests that SRT1 expression is not under glucose catabolite repression, or substrate induction, as might be expected, but is instead induced by signals from the plant, allowing it to be specifically expressed when required during the biotrophic growth of *U. maydis* within maize tissue. To test their hypothesis that Srt1 is necessary for sucrose degradation in plants, the authors first carried out a targeted deletion of *SRT1* and showed that it was necessary for virulence of the fungus. They then expressed an *Arabidopsis* sucrose transporter gene, *SUC9* [17], and showed that this was able to restore the ability of a *srt1* mutant to cause disease. When considered together, these results indicate that transport of sucrose by Srt1 is an essential requirement for the ability of this fungus to cause disease. As *Arabidopsis* SUC9 is unlikely to have additional functions within the fungus [17], it seems most likely that the role of Srt1 in planta is therefore specifically associated with its ability to transport sucrose.

Previous studies in pathogenic fungi have suggested a role for infection-specific hexose transporters in plant infection. The broad bean rust fungus *Uromyces fabae*, for instance, expresses a H+ symporter with specificity for glucose and fructose in its haustoria—the specialised invasive hyphae made by rusts and powdery mildews [19]. Electrophysiological studies showed its substrate specificity, whereas cytological analysis showed that it is very specifically localised at the haustorial plasma membrane. Similarly, the mycorrhizal fungus *Geosiphon pyriformis*, which forms a symbiotic relationship with plant roots allowing them to acquire nutrients more efficiently from soil, possesses a novel hexose transporter with highest affinity for glucose (followed by mannose, galactose, and fructose), which may be involved in the fungus–root cell mutualistic symbiosis that is of such importance to plant growth in diverse terrestrial ecosystems [20]. However, the role of these transporters in each respective plant–fungal interaction could not be determined by gene functional analysis because both *U. fabae* and *G. pyriformis* are obligate symbionts, which cannot be cultured away from their plant hosts, so the significance of these transporters to growth of each fungus in living plant tissue could not be directly assessed. For the first time, Wahl et al. [15], have therefore provided direct evidence of the utilisation of sucrose by a plant pathogen, challenging a widely held view that sucrose utilisation by fungi is the result of extracellular invertase activity from the fungus followed by specific transport of glucose [21].

An additional strategic advantage of directly utilising sucrose from the plant is also apparent from this work. Recently, a number of studies have indicated that glucose signalling may be intimately connected with the elicitation of plant defence responses [22],[23]. By direct utilisation of sucrose from the apoplast, the fungus may be able to evade recognition much more effectively while at the same time taking advantage of the major carbon source available within plant tissue. How widespread this form of growth will prove to be is, at present, unclear. However, close homologues of Srt1 are found in a range of both biotrophicnd free-living fungi, including closely related biotrophic *Ustilago* species, which are also amenable to gene function analysis [15]. This enables a direct test of this hypothesis within a range of both plant pathogenic and saprotrophic species to determine the relevant contribution of Srt1 homologues to the ability of fungi to grow either as pathogens, or saprotrophically. In this way it may be possible to investigate the evolution of sucrose transporters in fungi and shed light on whether biotrophic pathogens, such as *U. maydis*, have evolved specific mechanisms to allow utilisation of carbon substrates from living tissue that limit their ability to be detected by the host and whether this contrastswith necrotrophic and free-living fungal species.

The current study allows the formulation of a model by which *U. Maydis* is able to acquire apoplastic sucrose via the activity of a very high-affinity sucrose transporter that is present specifically in the fungal plasma membrane and, by means of its high-substrate specificity and extremely high affinity for sucrose, is able to efficiently out-compete plant apoplastic sucrose transporters and the presence of plant extracellular invertases and plant membrane–localised hexose transporters, thereby allowing efficient growth and development of the plant pathogen while preventing the induction of plant defences.
